# Comparison of trait and state mind wandering among schizotypal, subclinically depressed, and control individuals

**DOI:** 10.1186/s12888-024-05871-4

**Published:** 2024-06-05

**Authors:** Ya Wang, Tao Chen, Ji-fang Cui, Jia-li Liu, Tian-hong Li, Tian-jiao Du

**Affiliations:** 1 School of Psychology, Capital Normal University, Baiduizi 23A, Haidian District, Beijing, 100073 China; 2 Brain and Mind Centre, The University of Sydney, Sydney, Australia; 3 School of Psychology, The University of Sydney, Sydney, Australia; 4 Institute of Educational Information and Statistics, National Academy of Educational Sciences, Beijing, China; 5 Neuropsychology and Applied Cognitive Neuroscience Laboratory, CAS Key Laboratory of Mental Healthy, Institute of Psychology, Beijing, China; 6 Department of Psychology, University of Chinese Academy of Sciences, Beijing, China; 7 Department of Psychology, School of Medical Humanities, China Medical University, No.77 Puhe Road, Shenyang North New Area, Shenyang, Liaoning Province China

**Keywords:** Schizotypal, Depressed, Mind wandering, Daily life, Laboratory

## Abstract

**Background:**

Mind wandering is a common phenomenon in daily life. However, the manifestations and cognitive correlates of mind wandering in different subclinical populations remain unclear. In this study, these aspects were examined in individuals with schizotypal traits and individuals with depressive symptoms, i.e., subclinical populations of patients with schizophrenia and depression.

**Methods:**

Forty-two individuals with schizotypal traits, 42 individuals with subclinical depression, and 42 controls were recruited to complete a mind wandering thought sampling task (state level) and a mind wandering questionnaire (trait level). Measures of rumination and cognitive functions (attention, inhibition, and working memory) were also completed by participants.

**Results:**

Both subclinical groups exhibited more state and trait mind wandering than did the control group. Furthermore, individuals with schizotypal traits demonstrated more trait mind wandering than individuals with subclinical depression. Rumination, sustained attention, and working memory were associated with mind wandering. In addition, mind wandering in individuals with subclinical depression can be accounted for by rumination or attention, while mind wandering in individuals with high schizotypal traits cannot be accounted for by rumination, attention, or working memory.

**Conclusions:**

The results suggest that individuals with high schizotypal traits and subclinical depression have different patterns of mind wandering and mechanisms. These findings have implications for understanding the unique profile of mind wandering in subclinical individuals.

**Supplementary Information:**

The online version contains supplementary material available at 10.1186/s12888-024-05871-4.

## Introduction

### Mind wandering

Mind wandering refers to situations where the mind wanders away from the present task, and it is a phenomenon that occurs widely in daily life [1]. Mind wandering is thought to have negative consequences; for example, it impairs attention performance, can lead to accidents while driving, and may cause negative moods [1, 2]. Moreover, psychiatric patients showed a substantially altered frequency of mind wandering [3, 4]. Therefore, to better treat psychiatric disorders, it is important to achieve a deeper understanding of how mind wandering occurs in such populations.

### State and trait mind wandering

Mind wandering can be measured in different ways, such as questionnaires and thought sampling during experimental tasks. It is generally accepted that daily life mind wandering measured by questionnaires reflects the trait level and that mind wandering in the laboratory reflects the state level [5–7].

Seli et al. [5] found that state and trait levels of mind wandering were significantly correlated and validated each other. Studies have also demonstrated distinctions between trait and state mind wandering. For example, Kane et al. [8] suggested that mind wandering in daily environments and during controlled laboratory tasks has different correlates and may have different causes. Furthermore, state and trait mind wandering were differentially associated with motivation: state mind wandering was associated with state motivation and trait mind wandering was associated with trait motivation [7]. Therefore, it is necessary to examine both aspects of mind wandering.

### Mind wandering in psychiatric disorders

Patients with neuropsychiatric disorders (e.g., depression and schizophrenia) usually show elevated mind wandering. For example, patients with major depression have been found to engage in more mind wandering in daily life (trait level) and in the laboratory during a cognitive task (state level) [9–12]. During state mind wandering, individuals’ thoughts are more negative and less positive [9, 10].

In addition, patients with schizophrenia have also been found to exhibit a higher frequency of mind wandering both at the state and trait levels [13, 14]. One study showed the opposite findings, but this may be due to the low level of positive symptoms experienced by the patients in the study [15].

Mind wandering has been found to be associated with clinical symptoms in psychiatric patients, e.g., mind wandering enhanced depression, and was associated with positive symptoms in patients with schizophrenia [9, 13]. Therefore, studying mind wandering in these patients is important for understanding the psychopathology of psychiatric disorders.

### Mind wandering in subclinical populations

Neuropsychiatric disorders are considered a continuum, and there are subclinical populations between healthy individuals and clinical patients [16, 17]. Subclinical populations exhibited similar but milder degrees of psychiatric symptoms. Individuals with depression symptoms and individuals with schizotypal traits could be considered subclinical populations of patients with depression and schizophrenia. They also exhibited different profiles of mind wandering compared to healthy individuals.

Smallwood et al. [18] divided participants into two groups based on the mean depression score and found that the dysphoria group (those with high depression scores) demonstrated more state mind wandering than those in the low depression group during a word learning task and that they were more decoupled from the external environment during mind wandering. Marchetti et al. [9] reported that for individuals with higher levels of depression, state mind wandering during an attention task predicted greater accessibility of negative thoughts after the task.

Studies have also examined the relationship between mind wandering and depression in the general population. Vannucci et al. [19] reported that the frequency of trait mind wandering was positively associated with depressive symptoms. Studies measuring state mind wandering in the laboratory by thought sampling probes embedded in the sustained attention to response task (SART) revealed that depressive symptoms were positively correlated with the frequency of mind wandering [20, 21]. The results from the abovementioned studies suggest that depression is associated with increased state and trait levels of mind wandering.

Several studies have also examined the association between schizotypal traits and mind wandering. For example, Yamaoka and Yukawa [22] reported that the total score on the Schizotypal Personality Questionnaire (SPQ) was positively associated with trait mind wandering frequency measured by a questionnaire. Kane et al. [23] reported that positive, disorganized, and paranoid schizotypal traits were positively associated with state mind wandering measured by thought probes during cognitive tasks.

In addition, Zhang et al. [24] screened individuals with schizotypal traits and reported a higher frequency of trait mind wandering in these individuals than in the control group. However, few studies have examined state mind wandering using thought sampling tasks in individuals with schizotypal traits. Moreover, state mind wandering can be further divided into several dimensions.

### Meta-awareness and intentionality of mind wandering

In laboratory-based thought sampling tasks, other dimensions of state mind wandering in addition to frequency, such as meta-awareness and intentionality, were also studied. In a typical thought sampling task, participants performed a cognitive task, and random probes were used to ask whether the participants were focused on the task or off task (i.e., mind wandering) immediately before the probe appeared. If the participants were mind wandering, then they were further asked whether they were aware of their off-task status before the probe (meta-awareness) and whether they were mind wandering intentionally (intentionality) [25, 26].

Studies have suggested that healthy participants have more mind wandering with meta-awareness than without meta-awareness and have more unintentional than intentional mind wandering [27]. In the general population, depression was associated with mind wandering without meta-awareness (zone out) but not with mind wandering with meta-awareness (tune out) [20]. However, Nayda and Takarangi [21] reported that both mind wandering with and without awareness were positively associated with depression. In addition, depression was associated with unintentional (but not intentional) mind wandering [19, 28]. Therefore, it is necessary to differentiate these dimensions. Moreover, several factors are associated with mind wandering and may underlie the abnormalities of mind wandering in psychiatric patients and subclinical populations.

### Factors related to mind wandering

Rumination is a form of repetitive self-focused thought and usually increases depressive symptoms [29]. During rumination, individuals usually think about negative themes that direct attention away from the current task [30]. Therefore, there are some similarities between rumination and mind wandering, although differences also exist: for example, thoughts during mind wandering are free to move from one topic to the next, while thoughts during rumination tend to remain on a single theme [31].

Depression is strongly associated with rumination [32], and schizophrenia is also related to greater rumination [33]. It has been suggested that rumination plays an important role in the relationship between depression and mind wandering [21, 30, 34]. Individuals with depression tend to ruminate, which makes their minds wander more often [21]. This is particularly true for mind wandering without meta-awareness; if the participants were ruminating on negative things and were not aware of these things, then their mood state will deteriorate [21].

In addition, mind wandering was proposed to denote a failure of the executive control process as the mind wanders from the primary task [35, 36]. Indeed, participants with lower executive ability, such as reduced working memory capacity, were associated with a greater propensity for mind wandering [37, 38]. Participants with lower sustained attention ability and poorer response inhibition were also found to report more mind wandering [39, 40]. However, few studies have examined the role of cognitive processes in mind wandering in subclinical populations.

### The present study

Given the above, there are several limitations among previous studies. First, no study has compared mind wandering in individuals from different subclinical populations. The Research Domain Criteria (RDoC) encourages transdiagnostic comparisons to obtain a clear picture of the unique characteristics of specific disorders and to help clarify the underlying mechanisms [41]. It is suggested that both individuals with schizotypal traits and depressed individuals are more vulnerable to the influence of personal current concerns and affective dysregulation on their thought; therefore, they would be expected to show more mind wandering [9, 23]. In addition, schizophrenia and depression patients have differential abnormalities in the default mode network and executive control network [42], and the default mode network and executive control network are involved in mind wandering [43]. Therefore, schizophrenia and depression may have different patterns of mind wandering. Corresponding interventions may be developed based on these findings. Transdiagnostic comparisons could also be extended to subclinical populations [44, 45]. By comparing individuals with schizotypal traits and subclinically depressed individuals, one can better understand the unique characteristics of mind wandering in each group. Second, only a few studies have examined both state and trait mind wandering. Given the difference between state and trait mind wandering [7], it is necessary to examine both aspects in one study.

Therefore, the aim of the present study was to examine both state (measured by a laboratory thought sampling task) and trait (measured by a questionnaire) mind wandering among individuals with schizotypal traits and those with subclinical depression. We also examined the associations between mind wandering and rumination and executive abilities and further examined the group differences in mind wandering after controlling for correlated factors.

We hypothesized that both individuals with schizotypal traits and individuals with subclinical depression would exhibit increased state and trait mind wandering and that mind wandering would be positively associated with rumination and negatively associated with cognitive abilities. Furthermore, we predicted that group differences might be attenuated after controlling for these variables. Given that no study has directly compared different subclinical groups on mind wandering, we did not make specific hypotheses between the two subclinical groups.

## Method

### Participants

A total of 1,945 university students were recruited to complete online questionnaires, including the Schizotypal Personality Questionnaire (SPQ) [46, 47] and Beck Depression Inventory (BDI) [48, 49]. Three groups of participants were screened to complete the experiments and other questionnaires. According to the manual of the SPQ [46], those who scored within the top 10% in the SPQ could be considered individuals with schizotypal traits, and those who scored below the mean could be considered controls [50]. For the BDI, individuals who scored 14 or above could be considered individuals with subclinical depression, and those who scored 6 or below could be considered individuals without depression [49].

In the present study, the schizotypal group had an SPQ score ≥ 41 and a BDI score ≤ 6, the subclinical depression group had a BDI score ≥ 14 and an SPQ score < 41, and participants in the control group had an SPQ score ≤ 26 and a BDI score ≤ 6. A total of 106 schizotypal participants, 58 subclinically depressed participants, and 837 controls were identified. All 58 subclinically depressed participants were contacted, and 42 agreed to participate in the study. We also recruited 42 schizotypal participants and 42 controls. All participants additionally fulfilled the following criteria: no history of psychiatric or neurological disorders, no history of drug/alcohol abuse/dependence, and volunteered to participate. Participants in the control group also reported no family history of psychiatric disorders. This study was approved by the ethics committee of the last author’s affiliated institution. Participants signed informed consent before the formal experiment.

### Measures

#### Schizotypal personality questionnaire (SPQ)

We used the SPQ [46, 47] to measure schizotypal traits. The SPQ is a 74-item yes/no questionnaire. A “yes” response is assigned a score of 1, and a “no” response is assigned a score of 0. A higher score indicates more schizotypal traits. The Chinese version was adopted in the present study [47], and it showed good psychometric properties.

### Beck depression inventory (BDI)

The Chinese version of the 21-item BDI [48, 49] was adopted to measure depressive symptoms. Each item consists of four statements (scored 0–3), and participants were required to choose the one that best fit them. A higher score indicates more severe depression. The Chinese version showed good psychometric properties [49].

### Mind wandering experiment (thought-sampling task)

Probes were embedded in the Sustained Attention to Response Task (SART) [51] to measure mind wandering in the laboratory setting (state mind wandering). The task has been described in detail in Chen et al. [15].

Briefly, in the SART task, each trial began with a mask presented for 900 ms, followed by a digit (1–9) presented on the screen for 250 ms. Participants were required to press the left button of the mouse for nontargets (1–2, 4–9) and withhold the response for targets (3). There were 720 trials divided into 16 blocks of 45 trials. Digits were randomly presented. Each block included a thought probe located at a random place. For each probe, participants were first asked to indicate whether they were on task or not immediately before the probe, and they had four choices: (1) On task: The participants focused on performing the task; (2) Task-related interference: Participants thought about something related to the task, such as “How did I perform on this task?”; (3) External disturbance: Participants were distracted by external stimuli, such as noise outside the laboratory; and (4) Mind wandering: Participants thought about something unrelated to the task and were stimulus-independent [52].

These categories were explained to the participants, and they confirmed that they understood the categories before the formal experiment. If the participants were mind wandering, then they were asked to write down their thoughts briefly on the piece of blank paper given to them before the experiment. Then, they were asked to indicate whether they were already aware that they were mind wandering before the probe (meta-awareness) and whether they were mind wandering intentionally or unintentionally (intentionality). There was a practice block before the formal experiment. Go accuracy, correct Go reaction time, and No Go accuracy on the SART were taken as measures of sustained attention.

### Mind wandering questionnaire (MWQ)

The Chinese version of the 12-item MWQ [53, 54] was adopted to measure trait mind wandering. Participants rated each item on a five-point scale, with a higher score indicating more frequent mind wandering in daily life. The Chinese version showed good psychometric properties [54].

### Rumination response scale (RRS)

The Chinese version of the RRS [55, 56] was adopted to measure rumination thoughts. It is a 22-item questionnaire with a four-point rating (1 = never; 4 = always), and a higher score indicates more ruminative thinking. The Chinese version showed good psychometric properties [56].

### Flanker task

This task was adopted to measure cognitive inhibition [57]. In each trial, a fixation (“+”) was presented on the screen for a duration randomly selected from 800, 1,000, and 1,200 ms, followed by standard arrows (four peripheral arrows without the central arrow, e.g., >> >>, << <<) presented for 100 ms. Then, the central arrow appeared either compatible or incompatible with the peripheral arrows (e.g., <<<<<, <<><<). This slide was presented for no more than 1,600 ms and disappeared after a response was made. Participants were required to judge the direction of the arrow in the center by pressing “n” or “m” on the keyboard with their right hand. There were 64 trials in the formal experiment: half were compatible and half were incompatible. Participants had a practice block of ten trials before the formal experiment. The differences in reaction time and accuracy between incompatible and compatible trials (flanker effect) were indicators of cognitive inhibition.

### Chinese letter-number span (CLN)

The CLN [58] was adopted to measure working memory capacity. The CLN is similar to the English version of the Letter-Number Span task. Gan-Zhi names, which had an innate order as English letters, were mixed with digits and read to participants. Participants were required to say the digits first from small to large and then Gan-Zhi names in their innate order. Each length (the total number of digits and Gan-Zhi names) had four items. The test stopped when all four items of the same length were wrong. The total number correct and the longest span passed were recorded as working memory performance.

### Procedure

The SPQ and BDI were used to screen participants. Participants eligible for the experiment were invited to the laboratory to undergo the mind wandering experiment and then undergo other measures in a random order.

### Data analyses

Demographic variables and the SPQ and BDI scores were compared using one-way ANOVA or the *χ*^*2*^ test. Self-reported measures (trait mind wandering, rumination), mind wandering experimental measures, and other cognitive function measures (SART measures, flanker effect, working memory capacity) were compared between groups using one-way ANOVA, and pairwise comparisons (Bonferroni corrected) were also conducted. To examine the associations between mind wandering and other variables, we conducted Pearson correlation analysis for all participants (this analysis was also performed for each group, and the results are presented in Supplementary Materials Table S1). We further compared group differences in mind wandering, controlling for the variables that showed significant associations with mind wandering. The significance level was set at *p* < 0.05 unless otherwise indicated.

## Results

### Demographic information of participants and self-report measures

There were no significant differences in sex ratio or age among the three groups. There were significant group differences in the SPQ score and BDI score, suggesting the validity of group division.

The main effect of group on the mind wandering questionnaire score was significant (F(2,122) = 16.75, *p* < 0.001, *η*_*p*_^*2*^ = 0.215), and pairwise comparisons revealed that individuals with schizotypal traits showed more trait mind wandering than did subclinically depressed individuals (*p* = 0.043, Cohen’s *d* = 0.58), who showed more trait mind wandering than those in the control group (*p* = 0.004, Cohen’s *d* = 0.69). There was a significant group effect on rumination (F(2, 122) = 36.76, *p* < 0.001, *η*_*p*_^*2*^ = 0.376). Pairwise comparisons revealed that both individuals with schizotypal traits (*p* < 0.001, Cohen’s *d* = 1.67) and those with subclinical depression (*p* < 0.001, Cohen’s *d* = 1.66) experienced greater rumination than those in the control group, but there was no significant difference between the schizotypal and subclinical depression groups (see Table 1).

**Table 1:** Demographic information of participants and self-report measures

	Schizotypal (*N* = 42)		Depressed (*N* = 42)		HC (*N* = 42)				Pairwise comparisons
	*Mean*	*SD*	*Mean*	*SD*	*Mean*	*SD*	*F/χ* ^*2*^	*p*	
Male: female	17 : 25		17 : 25		16 : 26		0.03	0.968	
Age (years)	18.17	0.62	18.31	0.56	18.17	0.54	0.87	0.424	
SPQ	42.38	3.34	27.62	7.68	11.52	4.04	346.98	< 0.001	Schizotypal > Depressed > HC
SPQ_cognitive-perceptual	17.43	5.00	12.31	4.46	6.19	3.42	70.44	< 0.001	Schizotypal > Depressed > HC
SPQ_interpersonal	19.45	4.53	12.31	5.10	3.76	2.70	144.32	< 0.001	Schizotypal > Depressed > HC
SPQ_disorganization	9.69	3.08	5.71	3.58	2.33	1.32	71.13	< 0.001	Schizotypal > Depressed > HC
BDI	7.31	3.86	20.02	8.14	0.83	1.01	146.03	< 0.001	Depressed > Schizotypal > HC
MW_questionnaire	41.88	6.32	38.02	6.88	32.85	8.09	16.75	< 0.001	Schizotypal > Depressed > HC
Rumination	48.31	8.25	48.88	9.01	34.95	7.75	36.76	< 0.001	Schizotypal = Depressed > HC

Note: HC = Healthy controls (Low SPQ and BDI scorers); SPQ = Schizotypal Personality Questionnaire; BDI = Beck Depression Inventory; MW_questionnaire = mind wandering measured by Mind Wandering Questionnaire. Pairwise comparisons were Bonferroni adjusted

### Mind wandering measured by thought sampling

Analyses of thought probes revealed that there was a significant group difference in total state mind wandering frequency (F(2, 123) = 7.24, *p* = 0.001, *η*_*p*_^*2*^ = 0.105). Pairwise comparisons revealed significantly more state mind wandering in individuals with schizotypal traits (*p* = 0.002, Cohen’s *d* = 0.82) and subclinical depression (*p* = 0.012, Cohen’s *d* = 0.66) than in individuals in the control group, with no significant difference between schizotypal and subclinical depression individuals. Group differences were significant for on-task frequency (F(2, 123) = 6.45, *p* = 0.002, *η*_*p*_^*2*^ = 0.095), and further analysis revealed that individuals with schizotypal traits exhibited lower on task frequency (*p* = 0.002, Cohen’s *d* = -0.78) than did controls. The main effect of group was significant for external disturbance (F(2, 123) = 3.54, *p* = 0.032, *η*_*p*_^*2*^ = 0.054), and individuals with schizotypal traits demonstrated more external disturbances than did controls (*p* = 0.027, Cohen’s *d* = 0.56). No significant group difference in task-related interference was found.

When state mind wandering was differentiated by meta-awareness, the main effect of group was significant for mind wandering with meta-awareness (F(2, 123) = 5.85, *p* = 0.004, *η*_*p*_^*2*^ = 0.087). Further analysis revealed that individuals with schizotypal traits reported more mind wandering with meta-awareness than those in the control group (*p* = 0.003, Cohen’s *d* = 0.72). There was also a significant group difference in mind wandering without meta-awareness (F(2, 123) = 4.10, *p* = 0.019, *η*_*p*_^*2*^ = 0.062), and pairwise comparisons demonstrated that subclinically depressed individuals reported more mind wandering without meta-awareness than those in the control group (*p* = 0.018, Cohen’s *d* = 0.56).

When state mind wandering was differentiated by intentionality, there was no significant group difference in intentional mind wandering, while there was a significant group difference in unintentional mind wandering (F(2, 123) = 6.85, *p* = 0.002, *η*_*p*_^*2*^ = 0.10). Further analysis demonstrated that both individuals with schizotypal traits (*p* = 0.006, Cohen’s *d* = 0.73) and those with subclinical depression (*p* = 0.004, Cohen’s *d* = 0.73) reported more unintentional mind wandering than those in the control group (see Table 2). The distributions of these data are presented in Fig. S1-S10 of the Supplementary Materials.

**Table 2:** Mind wandering in the thought sampling task in three groups

	Schizotypal (*N* = 42)		Depressed (*N* = 42)		HC (*N* = 42)				Schizotypal vs. HC	Depressed vs. HC	Schizotypal vs. Depressed
	*Mean*	*SD*	*Mean*	*SD*	*Mean*	*SD*	*F*	*p*	*p*	*p*	*p*
MW thought sampling	3.62	2.39	3.31	2.43	1.88	1.82	7.24	**0.001**	**0.002**	**0.012**	1.000
On task	5.90	3.53	6.86	3.83	8.79	3.87	6.45	**0.002**	**0.002**	0.060	0.739
External disturbance	3.60	2.16	3.00	2.06	2.31	2.41	3.54	**0.032**	**0.027**	0.469	0.663
Task related interference	2.88	2.14	2.83	2.08	3.02	2.76	0.08	0.928	1.000	1.000	1.000
MW with meta-awareness	2.95	2.35	2.07	1.87	1.48	1.70	5.85	**0.004**	**0.003**	0.519	0.134
MW without meta-awareness	0.67	1.05	1.24	1.90	0.40	0.94	4.10	**0.019**	1.000	**0.018**	0.172
Intentional MW	1.24	2.10	0.88	1.17	0.79	1.26	0.97	0.382	0.567	1.000	0.898
Unintentional MW	2.38	1.97	2.43	2.10	1.10	1.49	6.85	**0.002**	**0.006**	**0.004**	1.000

Note: HC = healthy controls; MW = mind wandering. The significant *p* values were bolded. Pairwise comparisons were Bonferroni adjusted

### Cognitive performance

There was a significant main effect of group on SART Go accuracy (F(2, 123) = 3.64, *p* = 0.029, *η*_*p*_^*2*^ = 0.056), and subclinically depressed individuals showed reduced accuracy compared to the control group (*p* = 0.025, Cohen’s *d* = -0.58). The group difference was significant for the Chinese Letter-Number Span total score (F(2, 123) = 4.37, *p* = 0.015, *η*_*p*_^*2*^ = 0.066). Further analysis demonstrated that the schizotypal group had a lower total score than the control group (*p* = 0.012, Cohen’s *d* =-0.65). There were no significant group differences in other SART, CLN, or Flanker task measures (see Table 3).

**Table 3:** Cognitive performances in the three groups

	Schizotypal (*N* = 42)		Depressed (*N* = 42)		HC (*N* = 42)				Schizotypal vs. HC	Depressed vs. HC	Schizotypal vs. Depressed
	*Mean*	*SD*	*Mean*	*SD*	*Mean*	*SD*	*F*	*p*	*p*	*p*	*p*
SART_Go_acc	0.95	0.07	0.93	0.09	0.97	0.04	3.64	**0.029**	0.842	**0.025**	0.337
SART_NoGo_acc	0.38	0.18	0.39	0.19	0.42	0.20	0.59	0.557	0.920	1.000	1.000
SART_Go_RT	344.32	53.57	338.08	75.48	349.50	52.21	0.37	0.695	1.000	1.000	1.000
Flanker_effect_acc	-0.07	0.08	-0.07	0.08	-0.06	0.07	0.24	0.786	1.000	1.000	1.000
Flanker_effect_RT	97.04	32.57	103.43	47.57	90.84	44.77	0.92	0.400	1.000	0.530	1.000
CLN_total	19.60	3.60	20.79	4.56	22.45	5.06	4.37	**0.015**	**0.012**	0.266	0.668
CLN_longest	7.40	1.48	7.67	1.32	8.05	1.38	2.26	0.109	0.110	0.639	1.000

Note: SART = sustained attention to response task; acc = accuracy; RT = reaction time; CLN = Chinese Letter-Number Span; HC = healthy controls. The significant *p* values were bolded. Pairwise comparisons were Bonferroni adjusted

### Associations between mind wandering and cognitive performance

Given the multiple correlations conducted, we considered those with *p* < 0.01 to be significant. The results revealed that trait mind wandering (measured by questionnaire) was significantly correlated with total state mind wandering in the laboratory (measured by thought sampling) (*r* = 0.34, *p* < 0.001); in addition, state mind wandering was also correlated with state mind wandering with meta-awareness (*r* = 0.32, *p* < 0.001) and unintentional state mind wandering (*r* = 0.27, *p* < 0.01). Rumination was associated with trait mind wandering (*r* = 0.48, *p* < 0.001). SART Go accuracy was negatively associated with total state mind wandering (*r* = -0.26, *p* < 0.01), state mind wandering with meta-awareness (*r* = -0.23, *p* < 0.01), and intentional state mind wandering (*r* = -0.38, *p* < 0.001). The CLN total number correct was negatively associated with state mind wandering with meta-awareness (*r* = -0.23, *p* < 0.01) (see Table 4).

**Table 4:** Correlation between mind wandering and cognitive variables in all participants

	MW_thought sampling	MW with meta-awareness	MW without meta-awareness	Intentional MW	Unintentional MW	MW_questionnaire
MW_questionnaire	0.34***	0.32***	0.10	0.18	0.27**	-
Rumination	0.19	0.10	0.16	0.05	0.19	0.48***
SART_Go_acc	-0.26**	-0.23**	-0.09	-0.38***	-0.01	-0.06
SART_NoGo_acc	-0.10	0.02	-0.19	-0.01	-0.11	0.02
SART_Go_RT	-0.10	-0.02	-0.13	0.01	-0.12	0.05
Flanker_effect_acc	0.09	0.14	-0.06	0.08	0.05	-0.01
Flanker_effect_RT	0.05	0.07	-0.02	0.10	-0.02	-0.09
CLN_total	-0.18	-0.23**	0.05	-0.15	-0.09	-0.21
CLN_longest	-0.11	-0.13	0.01	-0.07	-0.08	-0.14

Note: MW = mind wandering; SART = sustained attention to response task; acc = accuracy; RT = reaction time; CLN = Chinese Letter-Number Span. Given the multiple comparisons, we consider those with *p* < 0.01 as significant. ** *p* < 0.01; *** *p* < 0.001

When we compared group differences in mind wandering after controlling for variables significantly associated with mind wandering, we found that when rumination was controlled for, trait mind wandering still significantly differed among the three groups (*p* = 0.004, *η*_*p*_^*2*^ = 0.088); however, the subclinically depressed group no longer showed more trait mind wandering than the control group (*p* = 0.999). The group difference in state mind wandering without meta-awareness became nonsignificant (*p* = 0.089, *η*_*p*_^*2*^ = 0.039). Other results remained unchanged.

When SART Go accuracy was controlled for, the group difference in state mind wandering was still significant (*p* = 0.004, *η*_*p*_^*2*^ = 0.089); however, subclinically depressed individuals no longer showed more state mind wandering than individuals in the control group (*p* = 0.068). Other results remained unchanged.

When the CLN total number correct was controlled for, the results for all indices related to state mind wandering remained unchanged, while for the results on trait mind wandering, the difference between individuals with schizotypal traits and subclinically depressed individuals became nonsignificant (*p* = 0.062).

## Discussion

To our knowledge, this is the first study to examine mind wandering in different subclinical groups. The main findings were as follows: (1) Both individuals with schizotypal traits and subclinically depressed individuals exhibited more state and trait mind wandering, and individuals with schizotypal traits tended to show more trait mind wandering than subclinically depressed individuals. Individuals with schizotypal traits exhibited more state mind wandering with meta-awareness than controls, while subclinically depressed individuals exhibited more state mind wandering without meta-awareness than controls. Furthermore, both individuals with schizotypal traits and subclinically depressed individuals exhibited more unintentional state mind wandering than controls. (2) State and trait mind wandering had different associations with rumination and cognitive functions. When rumination or sustained attention was controlled for, the difference between subclinically depressed individuals and controls on mind wandering varied. When working memory was controlled for, the difference between individuals with schizotypal traits and subclinically depressed individuals on trait mind wandering became nonsignificant.

### Group comparisons of mind wandering

Individuals with schizotypal traits exhibited increased state and trait mind wandering, and the results were consistent with those of previous studies [22, 23, 59, 60].

Fazekas [61] suggested that mind wandering and hallucinations share some similarities, such as spontaneous, transient, and relatively unconstrained nature; during these processes, individuals spontaneously disengaged their attention from external events and were accompanied by excessive preoccupation with the inner world. Therefore, hallucinations were proposed to be considered intensified forms of mind wandering that are produced by the same mechanisms [61]. Individuals with schizotypal traits exhibited more positive schizotypal (cognitive-perceptual) traits than did controls, which are attenuated forms of positive symptoms in schizophrenia; thus, individuals with high schizotypal traits reported a greater frequency of mind wandering.

Both a questionnaire study [19] and a daily life experience sampling study [62] suggested that depression and sadness were associated with increased daily life mind wandering. Using thought probes, studies [20, 21, 63, 64] have also shown that depressive symptoms are associated with elevated state mind wandering. Individuals with depression may have a greater level of rumination and more difficulty refocusing their attention from task-unrelated thoughts to ongoing tasks [21, 65]; this point will be further discussed below.

The present study further demonstrated that individuals with schizotypal traits reported more frequent trait mind wandering than did subclinically depressed individuals, since no previous study has compared subclinical groups, this finding needs replication, and the question of why differences were found only in daily life measures (trait level) but not in experimental measures (state level) also warrants further examination.

When differentiating state mind wandering based on meta-awareness, individuals with schizotypal traits exhibited more mind wandering with meta-awareness, and subclinically depressed individuals exhibited more mind wandering without meta-awareness than controls. The meta-awareness of mind wandering may be related to meta-cognition [21]. Individuals with schizotypal traits had an overall greater frequency of mind wandering, and they did not have meta-cognitive impairments on their performance [66]; therefore, they reported a greater frequency of mind wandering with meta-awareness. The finding that subclinically depressed individuals showed more mind wandering without meta-awareness was consistent with the findings of Deng et al. [20]. Mind wandering without meta-awareness may result in the activation of automatic processing and the induction of more negative moods [20, 21], which may be why subclinically depressed individuals have a greater frequency of mind wandering without meta-awareness. However, further studies are needed to determine whether the results could be replicated.

When state mind wandering was differentiated based on intentionality, both individuals with schizotypal traits and depressed individuals exhibited more unintentional mind wandering, possibly because overall unintentional mind wandering was more frequent than intentional mind wandering [27]. Therefore, intentional mind wandering was at a low level, and no significant difference was found between groups. Subclinical groups were more easily distracted by their internal thoughts or negative emotions, showing unintentional mind wandering [19, 28]. However, these results should be interpreted with caution since reporting intentional or unintentional mind wandering relies heavily on meta-cognition.

### Associations between mind wandering, rumination, and cognitive performance

Rumination is an important symptom of patients with depression and is also elevated in schizophrenia patients [32, 33]. Rumination has similarities and differences with mind wandering [31]. In the present study, rumination was significantly associated with daily life mind wandering but not laboratory mind wandering. Self-reported mind wandering and rumination may occur at the trait level, while laboratory mind wandering may occur at the state level [5]. Trait rumination may be associated with trait mind wandering, but the association with state mind wandering is small; further studies are needed to test this hypothesis.

Attention was significantly associated with laboratory mind wandering, which is aligns with the attentional resource theory [36, 67, 68]. These findings suggest that during a task, mind wandering may involve competing attention resources with performing the task. Furthermore, attention performance was associated with mind wandering with meta-awareness and intentional mind wandering, further suggesting that meta-awareness and intentionality require attention resources.

Working memory was only associated with mind wandering with meta-awareness, and these results suggest that individuals with greater working memory capacity were less likely to have mind wandering with meta-awareness. This may be because if they were aware of their mind wandering, then they would have adequate cognitive resources to refocus back to their task [69]. Consistent with previous studies, laboratory-based and daily life mind wandering have different correlates [37].

### Potential underlying mechanisms of mind wandering in subclinical individuals

To examine the underlying mechanisms of mind wandering in the subclinical participants, we controlled for variables that showed significant associations with mind wandering and examined group differences.

We found that in subclinically depressed individuals, when rumination was controlled for, trait mind wandering and state mind wandering without meta-awareness were not significantly different from those in controls. These findings are consistent with previous findings that mind wandering in subclinically depressed individuals is more past-oriented and more negative, and these thoughts are strongly related to rumination [34, 70, 71]. During rumination, an individual is lost in self-related thought, which is similar to mind wandering without meta-awareness [34, 72].

When attention was controlled for, laboratory mind wandering in subclinically depressed individuals was not significantly different from that in controls. Together with the finding that subclinically depressed individuals showed impairment in attention performance, these findings suggest that attention plays an important role in state mind wandering in subclinically depressed individuals and that the trait and state mind wandering of these individuals may involve different mechanisms.

When working memory was controlled for, the difference in daily life mind wandering between individuals with schizotypal traits and those with subclinical depression became nonsignificant, together with the finding that working memory capacity was impaired in individuals with schizotypal traits, suggesting that the difference in trait mind wandering between the two subclinical groups might be related to working memory capacity.

Individuals with schizotypal traits still demonstrated greater frequency of mind wandering both in daily life and in laboratory tasks after controlling for rumination, attention, or working memory, suggesting that each of these factors could not account for the higher frequency of mind wandering in these individuals. As suggested by Kane et al. [23], positive and paranoid symptom-related thoughts may be triggered during a task, which could play a role in the high frequency of mind wandering in individuals with schizotypal traits.

### Limitations

There are several limitations in this study. First, we did not measure the content of mind wandering, such as time orientation, emotion, or freedom of movement. Further studies may take these aspects of mind wandering into consideration. Second, individuals with schizotypal traits are heterogeneous and can be classified as having positive schizotypal traits or negative schizotypal traits. Further studies could recruit different subtypes of schizotypal groups. Third, the association between mind wandering and cognitive performance could be moderated by the cognitive load of the task [73]. The present study did not manipulate task load, and further studies should take this factor into consideration.

## Conclusions

Individuals with schizotypal traits and subclinical depression exhibited more mind wandering in daily life and in laboratory situations, and individuals with schizotypal traits tended to mind wander more than did individuals with subclinical depression in daily life. Mind wandering in individuals with subclinical depression can be accounted for by rumination or attention, while mind wandering in individuals with schizotypal traits cannot be accounted for by rumination, attention or working memory alone. These findings suggest that individuals with schizotypal traits and subclinically depressed individuals have different patterns of mind wandering and different underlying mechanisms. Further studies are needed to examine ways to increase adaptive mind wandering and decrease maladaptive mind wandering in these individuals.

### Electronic supplementary material

Below is the link to the electronic supplementary material.

Supplementary Material 1

## Data Availability

The data relating to this manuscript are available upon request from the corresponding author.

## Abbreviations

BDI: Beck Depression Inventory
CLN: Chinese Letter-Number Span
MWQ: Mind Wandering Questionnaire
RRS: Rumination Response Scale
SART: Sustained Attention to Response Task
SPQ: Schizotypal Personality Questionnaire (SPQ)
