# Supplementary material for: Comparison of trait and state mind wandering among schizotypal, subclinically depressed, and control individuals
Source: BMC Psychiatry. 2024 Jun 5;24:422. doi: 10.1186/s12888-024-05871-4 (PMC11151557; doi:10.1186/s12888-024-05871-4)
Supplement: Supplementary file 1 — Supplementary Material 1 [file 12888_2024_5871_MOESM1_ESM.docx]

**Supplementary materials**

**Table S1** Association between state mind wandering, trait mind wandering, and cognitive performance in each group

|  | MW_thought sampling | MW with meta-awareness | MW without meta-awareness | Intentional MW | Unintentional MW | MW_questionnaire |
| --- | --- | --- | --- | --- | --- | --- |
| **Schizotypal group** |  |  |  |  |  |  |
| MW_questionnaire | 0.44** | 0.40** | 0.09 | 0.23 | 0.29 |  |
| Rumination | 0.01 | -0.02 | 0.07 | -0.01 | 0.02 | 0.29 |
| SART_Go_acc | -0.37* | -0.40** | 0.05 | -0.60** | 0.20 | -0.16 |
| SART_NoGo_acc | -0.10 | -0.03 | -0.16 | -0.02 | -0.10 | 0.12 |
| SART_Go_RT | -0.02 | -0.03 | 0.02 | 0.08 | -0.11 | 0.23 |
| Flanker_effect_acc | -0.08 | -0.09 | 0.01 | -0.11 | 0.02 | -0.17 |
| Flanker_effect_RT | 0.03 | 0.10 | -0.16 | 0.16 | -0.13 | -0.14 |
| CLN_total | -0.22 | -0.24 | 0.04 | -0.16 | -0.09 | -0.12 |
| CLN_longest | -0.01 | -0.04 | 0.06 | 0.02 | -0.04 | 0.09 |
| **Depressed group** |  |  |  |  |  |  |
| MW_questionnaire | -0.05 | -0.04 | -0.02 | -0.11 | 0.00 |  |
| Rumination | -0.07 | -0.11 | 0.03 | 0.08 | -0.12 | 0.38* |
| SART_Go_acc | -0.24 | -0.24 | -0.07 | -0.41** | -0.05 | 0.16 |
| SART_NoGo_acc | -0.30 | -0.02 | -0.37* | -0.07 | -0.32* | -0.01 |
| SART_Go_RT | -0.23 | -0.09 | -0.21 | -0.02 | -0.26 | 0.00 |
| Flanker_effect_acc | -0.11 | -0.24 | 0.09 | -0.01 | -0.13 | 0.21 |
| Flanker_effect_RT | 0.02 | 0.03 | -0.01 | 0.07 | -0.02 | 0.01 |
| CLN_total | 0.12 | 0.01 | 0.15 | 0.02 | 0.13 | -0.19 |
| CLN_longest | 0.19 | 0.09 | 0.15 | 0.05 | 0.19 | -0.10 |
| **Control group** |  |  |  |  |  |  |
| MW_questionnaire | 0.37* | 0.29 | 0.21 | 0.25 | 0.24 |  |
| Rumination | 0.05 | -0.03 | 0.15 | -0.11 | 0.16 | 0.31* |
| SART_Go_acc | 0.15 | 0.16 | 0.00 | 0.09 | 0.10 | -0.13 |
| SART_NoGo_acc | 0.25 | 0.21 | 0.10 | 0.10 | 0.22 | 0.10 |
| SART_Go_RT | 0.12 | 0.11 | 0.02 | -0.06 | 0.19 | 0.09 |
| Flanker_effect_acc | -0.01 | -0.10 | 0.16 | -0.09 | 0.06 | 0.03 |
| Flanker_effect_RT | 0.03 | 0.08 | -0.07 | 0.08 | -0.03 | -0.26 |
| CLN_total | -0.27 | -0.29 | 0.00 | -0.24 | -0.14 | -0.03 |
| CLN_longest | -0.42** | -0.33* | -0.20 | -0.26 | -0.29 | -0.18 |

Note: MW = mind wandering; SART = sustained attention to response task; acc = accuracy; RT = reaction time; CLN = Chinese Letter-Number Span.

**Figure S1** Frequency of mind wandering in the thought sampling task

**Figure S2** On task frequency in the thought sampling task


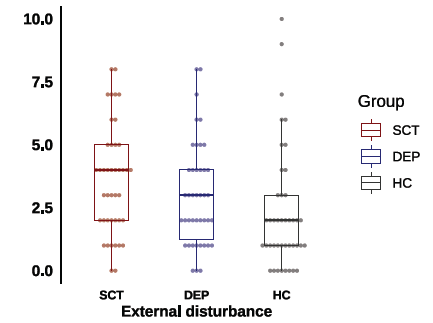


**Figure S3** External disturbance frequency in the thought sampling task


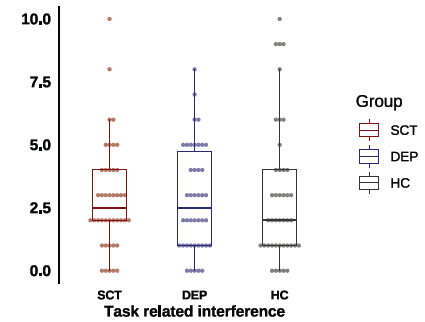


**Figure S4** Task related interference frequency in the thought sampling task


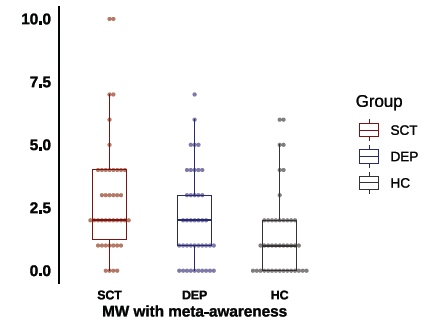


**Figure S5** Frequency of mind wandering with meta-awareness in the thought sampling task


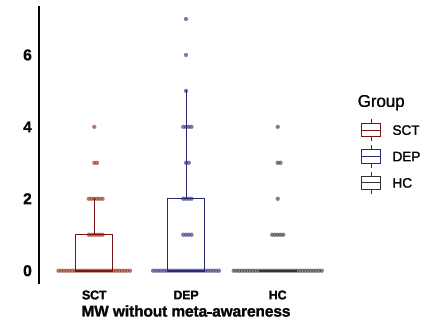


**Figure S6** Frequency of mind wandering without meta-awareness in the thought sampling task


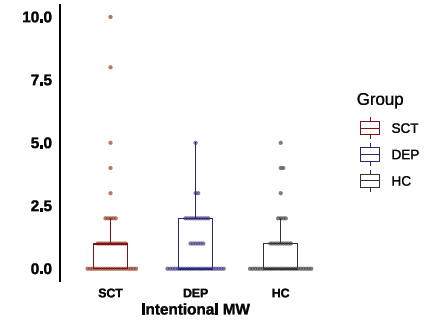


**Figure S7** Frequency of intentional mind wandering in the thought sampling task


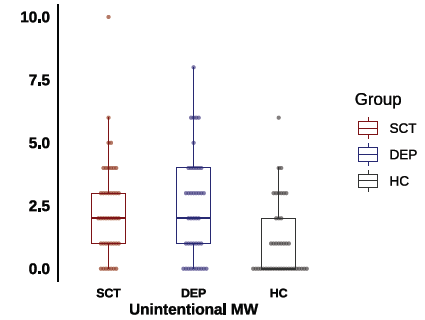


**Figure S8** Frequency of unintentional mind wandering in the thought sampling task

**Figure S9** Daily life mind wandering in the three groups


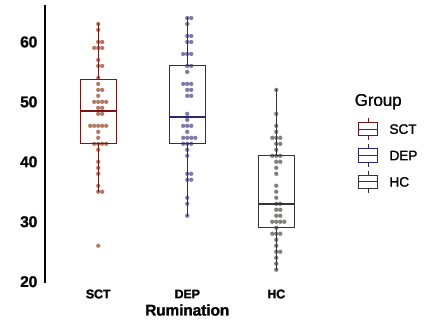


**Figure S10** Rumination in the three groups
